# Experimental Characterization of the Hydrodynamic Interactions between a Freely Rising Bubble and a Settling Particle

**DOI:** 10.1021/acs.langmuir.5c05905

**Published:** 2026-01-29

**Authors:** Masoud Outokesh, Mahdi Saeedipour, Mark W. Hlawitschka

**Affiliations:** † Institute of Process Engineering, 27266 Johannes Kepler University Linz, Altenbergerstr. 69, 4040 Linz, Austria; ‡ Department of Particulate Flow Modelling, Johannes Kepler University Linz, Altenbergerstr. 69, 4040 Linz, Austria

## Abstract

Bubble and particle interactions are fundamental in numerous industrial applications, particularly in the chemical and petrochemical industries, where three-phase reactors and slurry bubble columns are widely employed. Characterizing these interactions is inherently complicated as the mobility of the settling particle is coupled with the deformable nature of the rising bubble. This study attempts to unravel this complex system by developing a small-scale experimental approach to investigate and classify the different collision regimes. By utilizing a robust in-house image processing technique, we extracted the three-dimensional (3D) path of the particle during the interaction. A hydrodynamic force analysis method is applied to investigate the force balance exerted on the particle and the impulse variation during the interaction. Four distinguished regimes, called shuttling, bouncing, penetration, and flotation, are identified by the outcomes of the collision, based on hydrodynamic force balance. This approach can capture the transition between the different regimes at even higher particle concentrations or under different systematic parameters. These results provide fundamental insights into the bubble–particle interactions, offering a basis for developing scaled-up numerical models for the real-sized three-phase bubble columns.

## Graphical abstract

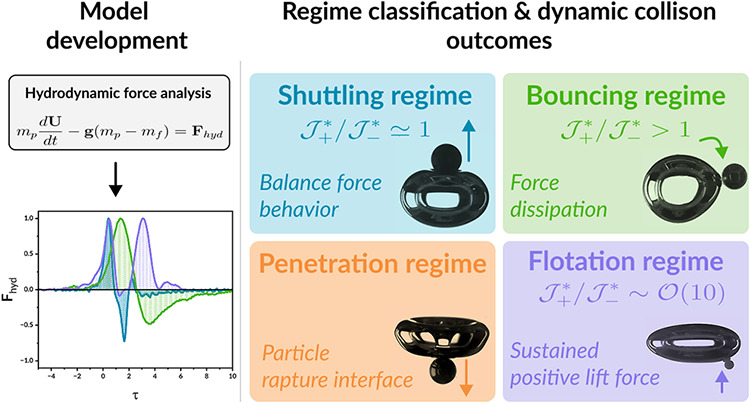

## Introduction

The interaction between bubbles and particles presents a complex multiphase phenomenon governed by a complicated interplay of hydrodynamic forces, interfacial tension, and surface chemistry. This fundamental understanding extends across a wide range of industrial sectors, including mineral processing,^1,2^ wastewater treatment,^3,4^ and chemical engineering.^5–7^ Within these applications, various complex three-phase (gas–liquid–particle) reactors are widely employed such as fluidized-bed reactors and slurry bubble column reactors (SBCRs). The behavior of these interactions between bubbles and particles is fundamental to describing the overall performance of such complex systems.^8^ In these columns, the presence of solid particles significantly alters the hydrodynamics, affecting gas holdup, bubble size distribution, liquid-phase mixing, and mass transfer rates.^9,10^ Consequently, a deeper investigation is required to define the bubble–particle interaction mechanism, which is essential to optimizing the design and operation of these crucial industrial processes. Such an in-depth analysis is possible with a small-scale scenario of bubble–particle binary interactions; one such scenario is the heads-on collision of one rising bubble and a settling particle.

The interaction between a settling particle and a rising bubble involves two different physics: the rigid settling surface of the particle and the deformable nature of the rising bubble interface, with which it collides. Particle settling is one of the fundamental phenomena that has been widely investigated in different situations.^11,12^ On the one hand, the falling motion of the particles is described by Newton’s second law, where there is a balance between the gravitational, buoyancy, and fluid dynamic drag forces.^13,14^ On the other hand, the behavior of the bubbles rising is a complex phenomenon that begins with an initial acceleration driven by buoyancy, going toward a constant terminal velocity as the upward buoyant force equilibrates with the combined downward forces of gravity and fluid dynamic drag.^15,16^ Furthermore, factors such as bubble size, shape, and interactions with walls can profoundly affect their shape, trajectory, and terminal velocity.^17–19^ While the interaction of rising bubbles with static rigid obstacles, such as walls,^20–23^ cylinders,^24–26^ and spheres,^27^ has been studied, the dynamic and unconstrained motion of settling particles introduces additional complexity, which requires more attention.

Although there are different cases and situations for studying the behavior of bubble and particle interactions,^28–34^ the literature is categorized into two main approaches based on the motion of the bubble. In the first method, known as the static approach, a bubble is generated and remains attached to the nozzle. Therefore, descending particles collide with the fixed interface of the bubble. In contrast, in the dynamic approach, the freely rising bubble interacts with moving settling particles. The static approach is designed occasionally to investigate the behavior of particles and bubbles during the flotation process. For instance, Brabcová et al.^35^ employed an experimental and theoretical method to analyze the trajectories of particles around a stationary bubble. Microhydrodynamic effects were found to play a crucial role in predicting the movement of particles around the bubbles. Different parameters, such as surface roughness, shape factor,^36,37^ and surface hydrophobicity,^38^ affect bubble–particle interactions.^39^ Hydrodynamic effects were studied under countercurrent flow conditions, showing that as the flow approaches the bubble, the direction shifts from axial to lateral. This increase in countercurrent flow rates enhances hydrodynamic drag, weakens radial particle motion, and affects attachment probabilities due to the limited drainage time of the liquid film. Yin et al.^40^ investigated bubble–particle collision, attachment, and detachment processes in fluidized-bed flotation using high-speed camera visualization and force measurements. Their results demonstrated that reducing particle settling velocity through rising water flow significantly increased bubble–particle contact time, thereby enhancing the probability of successful attachment and stable aggregate formation compared to conventional column flotation, where rapid settling prevented sufficient contact. These studies, primarily conducted under conditions of stationary bubbles, contrast with the more complex scenario of moving particles and bubbles, which presents significantly greater challenges due to the dynamic nature of both phases and the enhanced hydrodynamic complexity.

In the fully dynamic approach with a complete degree of freedom, the interaction between the settling particle and the rising bubble is considered. While some literature focuses on the dynamics of particles interacting with the fluid interfaces at a free surface,^41–43^ the bubble case is distinct because it includes additional forces, such as buoyancy. Hooshyar et al. experimentally investigated the hydrodynamic interactions between single rising bubbles and neutrally buoyant particles in liquid–solid suspensions.^44^ Two different interactions are introduced: indirect and direct bubble–particle interactions. The regime transition is characterized by a defined Stokes number $\left(\mathrm{St}=\frac{\tau_{p}}{\tau_{b}}\right)$ with τ_p_ representing the ratio of the particle relaxation time to the characteristic hydrodynamic time scale imposed by the rising bubble τ_*b*_. At low Stokes numbers (St. ≪ 1), particles follow liquid streamlines around the bubble without direct collision. At high Stokes numbers (St. ≫ 1), particle inertia leads to direct collisions, with energy transfer occurring through bubble deformation, resulting in a reduced bubble rise velocity governed by collision dynamics rather than viscous effects. Lyubimov et al. investigated the hydrodynamic aspect of these interactions in an incompressible viscous liquid subjected to ultrasonic vibrations.^45^ The influence of these vibrations acts as an attractive force, increasing the effective collision cross-section even with weak vibrations, indicating that solely considering monopole oscillations is inadequate for predicting bubble–particle interactions in oscillating flows. Additionally, the attachment process between the bubble and particle is studied using a numerical model by Je et al.^46^ The effects of parameters such as particle size and density were analyzed to determine the sliding time of the particle in this interaction. This remains independent of bubble size, leading to the development of novel probability models that decouple attachment into hydrodynamic and thermodynamic effects based on the particle Stokes number. Besides, the behavior of bubble–particle collision detachment in a semi-ideal settling condition is also investigated by Zhang et al.,^47^ and a three-dimensional detachment model is developed for bubble–particle aggregation based on these results. Furthermore, the hydrodynamic aspect of this interaction, in terms of the effects of varying Bond (gravitational/surface tension force) and Galilei numbers (gravitational/viscous force) on the formation and drainage of liquid films between the interfaces, was investigated numerically by Abdal et al.^48^ Low Bond numbers lead to the formation of a liquid film and drainage between the spherical bubbles and particles. While intermediate Bond numbers cause bubble sliding and detachment, high Bond numbers result in severe bubble deformation and rupture, allowing particle penetration. Simulation results also demonstrated that decreasing the particle-to-fluid density ratio enhances particle flotation by enabling bubbles to reverse the particle motion and lift them upward. At the same time, off-center collision produces sliding motions at low Bond numbers and tail formation at high Bond numbers, with contact line dynamics being significantly influenced by particle wettability, where hydrophilic particles promote strong bubble attachment compared to more hydrophobic surfaces.

While the existing literature has investigated various aspects of bubble–particle collisions under controlled conditions, further research is needed to classify and monitor collision outcomes in fully dynamic situations. In this study, the hydrodynamic interactions between a single rising bubble and a settling spherical particle are experimentally investigated in detail within a moderate range of fluid viscosities. A hydrodynamic force analysis method is developed to quantify different regimes and the inter-regime differences. Despite the simplifications in this small-scale approach compared with the haphazard interactions within SBCs, it still helps unveil the interaction between the rigid body of the settling particle and the deformable interface of the rising bubble. The article is structured as follows: Details of Experiments Section describes the experimental setup and the in-house multioperation image processing method, which is critical for accurately extracting the three-dimensional path of the particle. Next, the hydrodynamic force analysis method is introduced based on the instantaneous motion of the particle. Results and Discussion Section presents the experimental results, classifying the interaction into four distinct regimes. Additionally, analysis of the associated hydrodynamic force balance and impulse variations is performed for each regime

## Details of Experiments

### Experimental Setup

A centi-scale square cross-section (80 × 80 mm^2^) plastic column is constructed as demonstrated in Figure 1 for an experimental setup to observe the behavior of the bubble and particle interaction. In this setup, bubbles are generated by injecting air (Microlab 1000 precision pump) into needles of different sizes (8 and 15 gauge of Hamilton company needles), which are fixed at the bottom of the column. Additionally, the system of releasing particles also includes another needle at the top of the column, which is fixed to the optical table. Aligning the interaction between the bubble and the particle during the interaction is a complex and crucial process; therefore, an adjustable platform is constructed to line up their paths. To do so, the column is located on top of the tuning stage. This stage is a manual *XY* linear trimming platform that provides the ability to move the column location with an accuracy of 0.04% of the column width. Additionally, to ensure the accuracy of the interactions, any misalignment >5% between the bubble and particle center of mass in comparison to the bubble diameter is eliminated from the datasets in the postprocessing.

**Figure 1:**
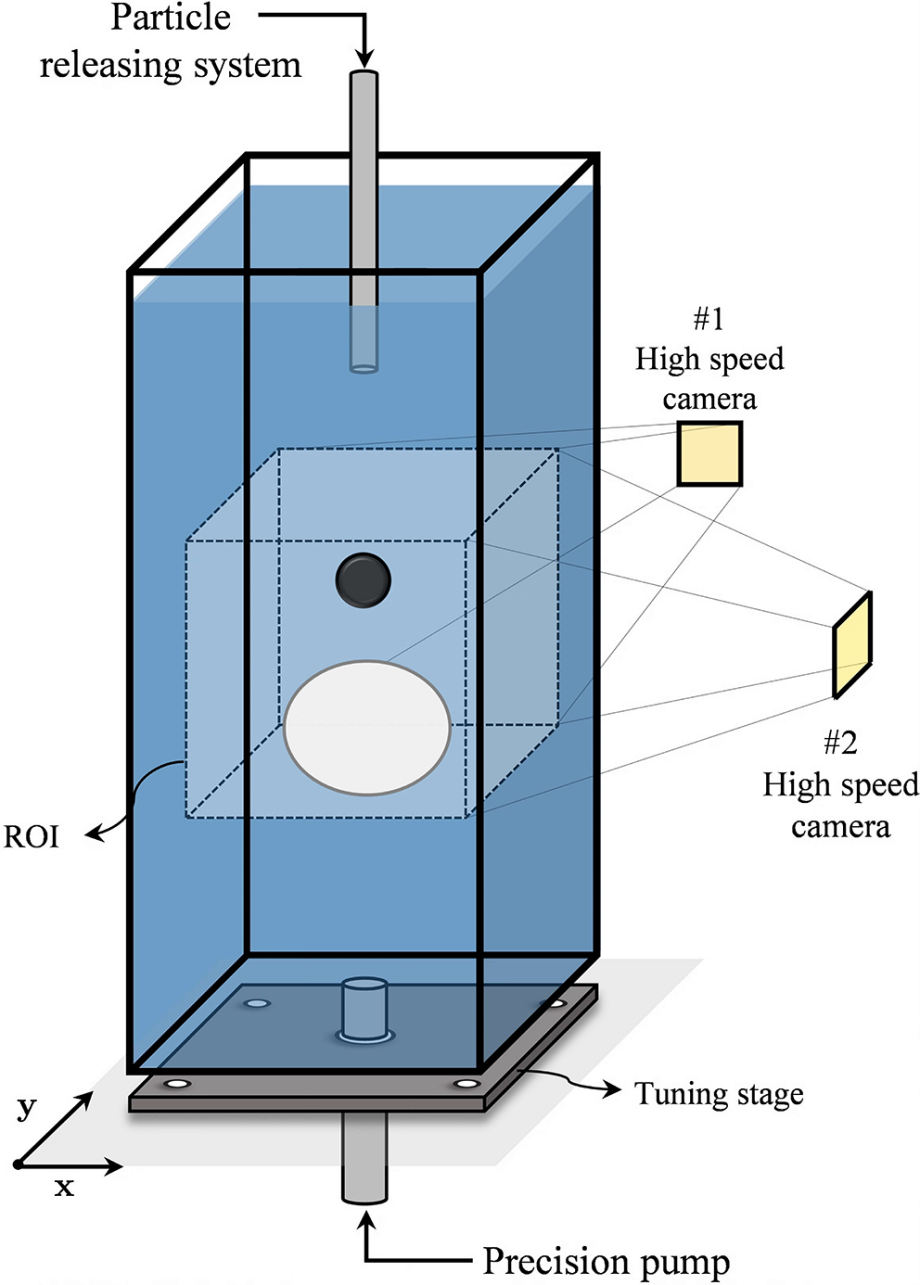
Schematic diagram of the experimental setup.

A pair of high-speed cameras (Integrated Design Tools Inc. (IDT), OS II – Series 8-S2), equipped with identical lenses (LAOWA, Super macro lens V-Dx 60 mm), is set up perpendicular to each other to capture the interaction from both perspectives. By capturing the high-frequency image sequence (2000 Hz) from two perpendicular sides, we can obtain different information about the nature of these interactions.

One of the important parameters that govern the interaction is the fluidic parameters of the medium fluid. By adjusting the glycerin–water weight ratio, various fluid properties can be achieved.^49^ All experimental measurements were performed under ambient room temperature conditions (22–23 °C). The viscosity of each fluid solution is measured using a ViscoQC 300 rotational viscometer, while the density is obtained with a DMA 35N portable density meter (Anton Paar GmbH, Graz, Austria). Surface tension is also estimated by the pendant drop method through open-source software called OpenDrop.^50^

### Postprocessing Method

A multioperation particle tracking system is developed to analyze the raw image sequence captured from the experimental section, to track particle movement in this complex system. Traditional shadowgraph approaches fail when particles become partially or completely masked by bubble interfaces, experience dramatic lighting variations, or exhibit low contrast against complex backgrounds. These limitations present significant challenges to achieving reliable automation in high-speed imaging applications, where manual tracking becomes unfeasible due to the overwhelming amount of data or lack of accuracy and precision. The multioperator detection framework enhances detection accuracy and enables automated analysis of particle dynamics through robust redundancy and adaptability. This approach significantly advances automated particle tracking by removing the need for manual intervention during occlusions and ensuring consistent detection across various experimental conditions, thereby enhancing the reliability and reproducibility of quantitative measurements in fluid dynamics and materials science.

The proposed approach for image processing operates through a structured multistage pipeline as illustrated in Figure 2a, where the methodology is divided into four distinct processing units.

**Figure 2:**
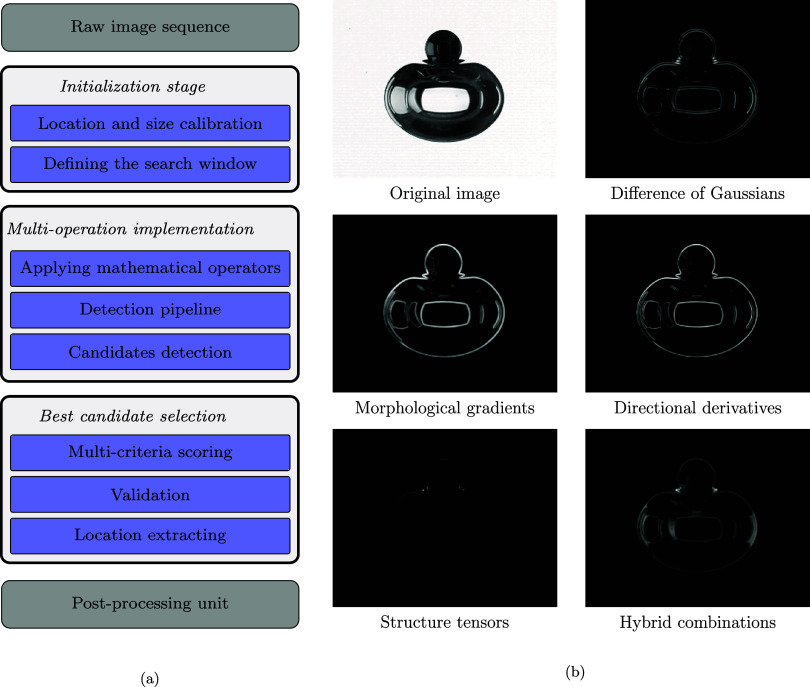
Multioperator particle tracking system. (a) Four-stage workflow diagram showing the systematic processing pipeline of the developed code. (b) Sample outputs from five mathematical operators applied to enhance particle detection under different computational principles.

The initialization stage includes calibrating location and size using the specified number of initial frames, followed by defining adaptive search windows centered on the previous detections of the center of the particle. During the multioperator implementation stage, six mathematical operators are applied simultaneously to each preprocessed frame. These operators are difference of Gaussians, morphological gradients, directional derivatives, structure tensors, and hybrid combinations. This is illustrated in Figure 2b, which displays representative outputs for each method. Each operator result then undergoes a detection pipeline encompassing Hough circle transforms, contour-based analysis, and arc detection algorithms specifically designed to handle varying degrees of particle occlusion. The best candidate selection stage employs multicriterion scoring that integrates detection confidence, raw image validation through contrast and edge alignment analysis, and temporal motion consistency checks to automatically identify the optimal detection from all operator–method combinations. Finally, in the postprocessing unit, parameters such as velocity or acceleration will be extracted based on the temporary location of the particle.

### Analysis Method

This study explicitly tracks the dynamics of settling particles and bases the analysis on the force balance during the descent. Consider the defined interaction window (Figure 3) that a falling particle with reached terminal velocity (*u*_p_) and predefined properties, including density (ρ_p_) and diameter (*d*_p_), interacts with a rising air bubble with specific density (ρ_b_) and viscosity (μ_b_), alongside the bubble terminal velocity (*u*_b_) and diameter (*d*_b_). The entire system operates within the fluid domain, characterized by specific density (ρ_f_), viscosity (μ_f_), and surface tension (σ) between the fluid and gas phases. The important nondimensional numbers of the parameters inside the system are the particle-to-bubble diameter ratio (λ = *d*_p_/*d*_b_) and the particle-to-fluid density ratio (Γ = ρ_p_/ρ_b_). Three primary fluids are selected to demonstrate the variation in regimes in this work. By introducing the viscosity ratio between the fluid viscosity and water viscosity μ* = μ_f_/μ_water_, the viscosity ratios for these systems are 22.5, 33.5, and 90. The surrounding fluid properties are further characterized by the Morton number (Mo = (ρ_f_ – ρ_b_) *g*μ_f_^4^/σ^3^ρ_f_^2^), with *log­(Mo)* values of −5.13, −4.43, and −2.71, respectively, as the viscosity ratio increases. The experimental parameters are gathered in Table 1.

**Figure 3:**
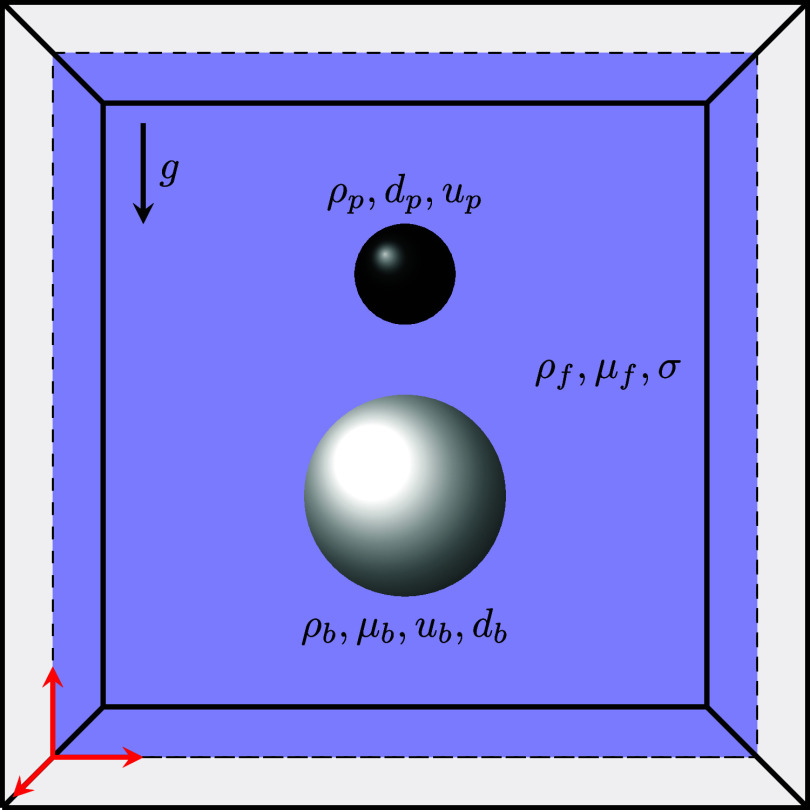
Schematic diagram of the region of interest.

**Table 1:** Experimental Parameters

items	value
particle diameter range, *d*_p_	0.7 – 2 [mm]
bubble diameter range, *d*_b_	4.44 – 4.87 [mm]
fluid viscosity range, μ_f_	22.5 – 90 [mPa.s]
fluid density range, ρ_f_	1175 – 1218 [kg/m^3^]
particle density range, ρ_p_	3200 – 7500 [kg/m^3^]

The instantaneous velocity of the bubble and the particle is measured based on their trajectories, which are achieved by tracking the centroid of their shape in each frame via two side cameras. Based on the bubble diameter and center-of-mass velocity, the bubble Weber number (We_b_) is defined as ρ_f_ *u*_b_^2^ *d*_b_/σ, which is the ratio of the inertia of the bubble to its surface tension force. In addition, the behavior of the particle inertia in the surrounding fluid is characterized by the particle Reynolds number (Re_p_ = ρ_f_ *u*_p_d_p_/μ_f_).

Particle movement in this system involves three main stages: (i) freely settling, (ii) interaction duration, and then (iii) settling again after collision. As a result, the motion of the particle is governed by a dynamic force balance in which the driving force, the net force of gravity and buoyancy, is opposed to and modified by hydrodynamic forces (including drag and lift) and short-range bubble–particle interaction forces as the particle approaches the bubble interface. This behavior is described by the classical Basset–Boussinesq–Oseen (BBO) force balance

1 $$m_{p}\frac{dU}{dt}=F_{\mathrm{wei}}+F_{\mathrm{buo}}+F_{\mathrm{am}}+F_{\mathrm{drag}}+F_{\mathrm{lift}}+F_{\mathrm{lub}}+F_{\mathrm{int}}$$

**U** represents the velocity of the center of mass of the particle. In addition, **F**_wei_ and **F**_buo_ define the forces exerted by the weight of the particle and buoyancy of the fluid, respectively. These two terms can merge as given below based on the mass of the particle (*m*_p_ = ρ_p_ π*d*_p_^3^/6) and displaced fluid mass (*m*_f_ = ρ_f_ π*d*_p_^3^/6)

2 $$F_{\mathrm{wei}}+F_{\mathrm{buo}}=g\left(m_{p}-m_{f}\right)$$

Moving on to other forces included in eq 1, the third term on the right-hand side represents the effects of particle motion in the fluid and its influence on accelerating the surrounding fluid, known as the added mass force (**F**_am_). This force becomes particularly important due to the relatively large acceleration during impact with the rising bubble. It is defined as

3 $$F_{\mathrm{am}}=C_{\mathrm{am}}m_{f}\frac{d}{dt}\left(U-V\right)$$

where *C*_am_ is the added mass coefficient and **V** is the fluid velocity. While *C*_am_ is assumed constant (0.5 for a single spherical particle in an unbounded fluid) during free settling, this coefficient may vary as the particle approaches the bubble interface, during the interaction, and in the postcollision phase due to the altered flow field.

Another important force that the fluid exerts on the particle due to the relative motion of the particle and the surrounding fluid is the viscous drag force. This force reflects the fluid resistance as a result of the particle’s movement. The general formulation of the drag force is defined as follows based on the projected area of the particle (*A*_p_ = π *d*_p_^2^/4) and the drag coefficient (*C*_D_)­

4 $$F_{\mathrm{drag}}=\frac{1}{2}C_{D}\rho_{f}A_{p}\left|U-V\right|\left(U-V\right)$$

In addition to drag, a lift force (**F**_lift_) may act on the particle when moving through a fluid with velocity gradients

5 $$F_{\mathrm{lift}}=C_{L}\rho_{f}V_{p}\left(U-V\right)\times \omega$$

where *C*_L_ is the lift coefficient, *V*_p_ is the particle volume, and **ω** is the fluid vorticity.

In addition to hydrodynamic contributions to particle settling, an interaction force (**F**_int_) arises once the particle comes into contact with or deforms the bubble interface. This force originates from surface tension, which acts to restore the bubble shape and can significantly influence rebound dynamics and energy dissipation.

The formation of a narrowing gap between the approaching bubble and particle generates a hydrodynamic pressure force (**F**_lub_), which is related to the thickness of the gap. As the gap thickness decreases, this resistance increases sharply and often governs the interaction by controlling the outcome of rebound or attachment. This force is related to the minimum thickness of the gap between two approaching interfaces (*h*(*t*)), the size ratio between the bubble and particle (λ), and the fluid viscosity (μ_f_).

Many of the mentioned forces require a velocity field inside the fluidic domain that is not achievable by the method used in this article. In addition, due to the limitations of optical measurement and the complexity of the collision between a rigid and a deformable body, the rapid shape deformation of the bubble interface remains unidentified. Therefore, all forces that depend on the relative velocity of the particle and the fluid domain, the interaction, and lubrication forces are merged into a term, hydrodynamic force (**F**_hyd_); then, eq 1 is rewritten as follows

6 $$m_{p}\frac{dU}{dt}-g\left(m_{p}-m_{f}\right)=F_{\mathrm{hyd}}$$

By utilization of the image processing method, the instantaneous location of the particle in each frame can be extracted. Therefore, the right-hand side of eq 6 is estimated, and the lump value of **F**_hyd_ is calculated in each time step.

As mentioned before, particle movement involves two main stages. In the freely settling particle, it achieves its equilibrium condition and the velocity reaches its terminal value. In this stage, |**F** _hyd_ | remains constant referred to as |**F**_hyd,eq_|, and the terms that are related to the collision with the bubble are eliminated from the right-hand side of eq 6. Instead, during the interaction, as the bubble approaches the particle, the equilibrium is disrupted, and interaction forces become dominant. The magnitudes of all forces involved in this interaction are monitored by comparing the conditions at each time step with the equilibrium condition. The force balance during the interaction is determined by subtracting the equilibrium force from the instantaneous hydrodynamic force defined as |**F**_hyd_^*^| = |**F**_hyd_|−|**F**_hyd,eq_|.

## Results and Discussion

Four regimes are classified based on the behavior of the bubble and particle during collision: shuttling, bouncing, penetration, and flotation. The distinguished behavior of the hydrodynamic force and the particle in the vicinity of the bubble is also investigated in each case.

### Shuttling Regime

The shuttling regime occurs when the bubble has the potential to reduce the inertia of the settling particle and act as a carrier, shuttling the particle over a considerable distance against gravity. Figure 4a, 4b illustrates the real snapshot and normalized location (x*, y*, z*) of the particle during a shuttling regime, showing that it is carried approximately 4.2 times the particle diameter upward (z* direction). This particular case occurs in the relatively high-viscosity domain *log­(Mo)* = −2.71), where elevated viscosity increases the drag coefficient for both the bubble and particle, thereby reducing their inertia and relative velocity. As the bubble deformation is more complex in a high-viscosity domain, it does not deform significantly during the interaction; instead, it acts like a rigid platform to lift the particle.

**Figure 4:**
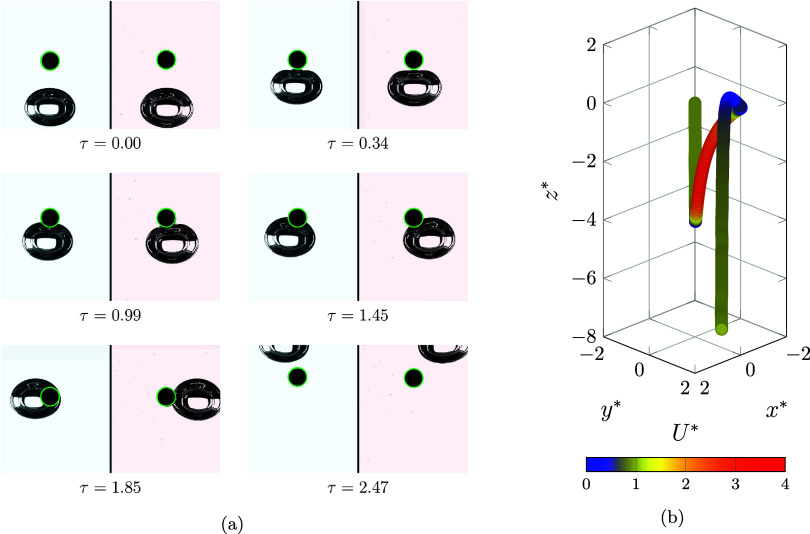
(a) Bubble and particle interaction in the shuttling regime (Γ = 2.62, λ = 0.41 and *log­(Mo)* = −2.71). (b) 3D path of the particle. Each component of the particle is normalized by the diameter of the particle (*d*_p_ = 2 mm). The color map demonstrates the normalized velocity magnitude of the particle (U*).

Even though the smaller size ratio between the particle and bubble (λ = 0.37) results in a larger ratio between the buoyancy of the bubble and the weight of the particle, |*F*_b,b_|/|*F*_g,p_| = 5.46, the dominance of viscosity helps the bubble control the interaction and prevent significant fluctuations. Moreover, higher viscosity reduces the inertia forces of both the bubble and the particle as the bubble Weber number and particle Reynolds number approach O ­(1). Consequently, the relative velocity between the bubble and the particle decreases.

For a better understanding of the particle behavior, a dimensionless time is defined as τ = *tu*_p_/*d*_p_. It should be noted that τ = 0 is set when the minimum distance between the bubble and particle interface reaches the diameter of the particle (*h* = *d*_p_), and therefore, both negative and positive τ values are allowed.

As demonstrated in Figure 4b, the particle continues on its straight path, while the velocity of the particle is the same as the terminal velocity. Then, the velocity starts to decrease due to the bubble being sensed. The displacement of the particle from the initial position ( $\Delta r=\sqrt{{\left(x^{*}-x_{0}^{*}\right)}^{2}+{\left(y^{*}-y_{0}^{*}\right)}^{2}+{\left(z^{*}-z_{0}^{*}\right)}^{2}}$ ) and the normalized velocity are shown in Figure 5a.

**Figure 5:**
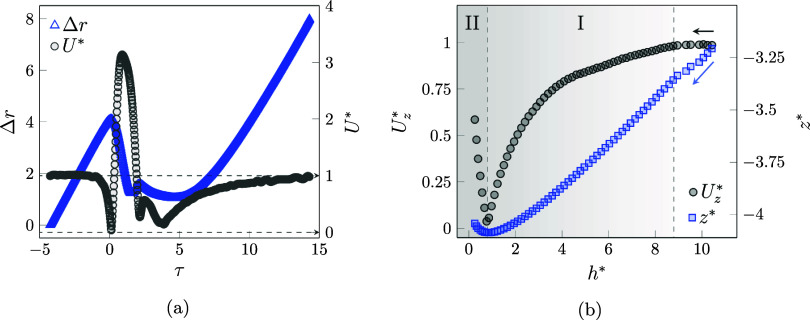
(a) Variation of the normalized displacement (Δ*r*) of the particle in the shuttling regime alongside the normalized velocity magnitude (U*). (b) Variation of the normalized velocity magnitude and the normalized *z*-direction location (z*) in the range of the normalized minimum distance between the bubble and the particle (h* = *h*/*d*_p_) before the collision.

The sequence begins with free particle settling, where a steadily increasing displacement is observed at a constant normalized velocity corresponding to the terminal velocity of the particle. Then, at τ = −0.80, it is observed that the velocity of the particle starts to decrease as it enters the surrounding environment of the rising bubble. Consequently, the rate of displacement decreases. At τ = 0.13, the bubble succeeds in completely stopping the particle (U* = 0.04) and changing its direction from settling to rising. As the particle shuttles with the bubble, the velocity magnitude increases sharply and matches the bubble velocity, which is affected by the additional weight (U* = 3.13 at τ = 0.89). At the same time, as the particle climbs roughly the same path it fell, the displacement decreases. Because of the inherent instability of this shuttling phenomenon, the particle begins to slide along the bubble interface. As a result, the velocity decreases; even as it was still climbing during the slipping, the displacement continued to decrease. The peaks and the variation in velocity and displacement over the duration 2.02 < τ < 3.77 are due to the particle being trapped in the wakes generated by the rising bubble.^51^ Even in this high-density ratio (Γ = 2.62) and viscous medium, these instabilities are able not only to lift the particle for a small distance but also to manipulate the velocity profile. After the particle is released from the region of the vortices, it again starts to settle and both displacement and velocity increase. The velocity magnitude tends to reach unity, which is equal to the condition of the particle settling with the terminal velocity.

The formation of a liquid film between the bubble and the object is reported in different studies in the cases of the wall,^52^ a static cylinder,^24,26^ and a freely moving particle.^48^ The variation of the normalized velocity magnitude and the normalized z position is monitored within the range of the normalized minimum distance between the bubble and particle interfaces (h* = *h*/*d*_p_, Figure 5b). Analysis of the motion of the particle reveals that its approach can be divided into two main stages. The behavior begins when the particle is positioned far from the bubble interface at large h*, where the value of the velocity is relatively constant; it can be assumed to be freely falling. Then, the slope of the decrease in velocity magnitude decreases as the particle senses the bubble closing. As a result, the velocity magnitude starts to decrease as the descending movement of the particle must be stopped (stage I). This stage starts from h* = 8.8 and shows that in such a high-viscosity fluid, the thick surrounding region has the potential to resist the particle inertia force. The impact area around the rising bubble also moves toward the particle and has the potential to influence the surrounding objects. When a particle enters this environment, the pressure inside the liquid film stops its downward motion. This pressure is determined by the distance between the interface and the particle, the relative velocity between the two objects, and the viscosity of the fluid. As the bubble and particle get closer, the bubble interface begins to deform. The liquid film pressure builds up, causing the particle to change direction and begin to climb, even though there is a distance of about 0.8 times the particle diameter between the two interfaces and the two interfaces do not touch. After this distance, the particle attempted to match its velocity with the rising bubble, and the second stage began until a touch occurred (stage II). Although the formation and acting of the liquid film are mentioned and investigated in the literature,^53^ there is a significant concern about exploring its behavior.

In order to investigate the forces that are exerted on the particle during this regime, the behavior of |**F**_hyd_^*^| in the range of the interaction is monitored. Therefore, Figure 6a demonstrates the behavior of the normalized hydrodynamic force with the maximum value of that over the entire time duration. By analyzing the behavior of the particle in relation to the displacement and the velocity (Figure 5a), it is possible to describe the behavior of the hydrodynamic interaction of this collision.

**Figure 6:**
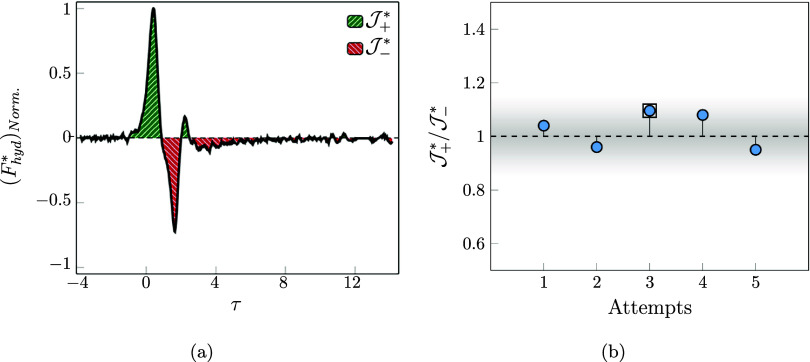
(a) Variation of the normalized hydrodynamic force in the shuttling regime. (b) Different values of the positive to negative impulse magnitude ratio ( $J_{+}^{*}/J_{-}^{*}$ ) in shuttling regime attempts. The described case with *log­(Mo)* = −2.71 is marked as attempt #3.

As the particle in the freely settling duration experienced a constant velocity, the balance forces that are exerted on the bubble become zero (eq 1). It is only the combination of the drag and buoyancy force in contrast to the weight of the particle. Then, the system is in an equilibrium condition. The moment the particle path is manipulated by the surrounding region of the bubble, this equilibrium condition is disrupted. Therefore, the role of the lubrication force in the balance of eq 1 starts to become more dominant. This force increases until the first peak (τ = 0.41), at which point the particle appears to settle on the bubble and a concave region forms at the apex of the interface, while, due to the disappearance of the particle kinematic energy via the lubrication force and the high viscosity of the fluid, the bubble aspect ratio does not experience a large deformation or oscillation. The force experienced during the peak in Figure 6a is 1.8 times the equilibrium force, and after the peak, as the acceleration of the particle decreases, the force magnitude decreases. As the particle matches the velocity of the bubble, the pressure inside the bubble increases due to the additional weight of the particle. The moment the particle starts to slide over the bubble, the hydrodynamic force reaches 0 (τ = 0.89). Then, as the particle starts to slide over the bubble, after a rounding motion (0.89 < τ < 1.30), the force drops below the equilibrium force. When the particle is located on the side of the bubble, the force starts to grow until it again reaches the value of the equilibrium condition (τ = 0.99).

From this moment, the particle is contained within the vortices of the bubble, and the positive disturbance occurring between 2.02 < τ < 3.77 is attributed to that. Then, the particle again starts to settle freely, and until the force reaches terminal velocity, the hydrodynamic force tends to reach equilibrium condition ((**F**_hyd_^*^)_norm._ = 0).

By integrating the instantaneous hydrodynamic force over a dimensionless time, the dimensionless impulse is defined ( $J^{*}$ ). The positive impulse impact is due to the particle stopping the downward motion and shuttling section. By comparing the positive and negative impulse magnitudes, a physical indicator is defined as $J_{+}^{*}/J_{-}^{*}$ , which varies around 1 for all the tested cases with shuttling as shown in Figure 6b.

### Bouncing Regime

When the force exerted by the particle is within the range of the surface tension force and the surrounding fluid viscosity is not able to resist the kinematic energy of the particle, the collision between the bubble and the particle occurs with higher intensity. The deformable nature of the bubble acts like a spring during the interaction, and the behavior is related to the capillary pressure inside the bubble, the surface tension at the interface, and the force ratio between the particle weight and bubble buoyancy force. When the kinematic energy of the particle is insufficient to penetrate the interface, the bubble absorbs that energy. Then, it returns a portion of it to the particle, causing the particle to bounce. Figure 7a illustrates a snapshot of the bouncing interaction regime from two perpendicular views in a moderate fluid viscosity (*log­(Mo)* = −4.43, Γ = 6.31, and λ = 0.33). A higher density ratio in this case increases the particle Reynolds number ( Rep≃O ­(10)), and the ratio of bubble to particle terminal velocity approaches unity (*u*_b_/*u*_p_ = 1.03), which means a higher relative velocity between bubble and particle interfaces.

**Figure 7:**
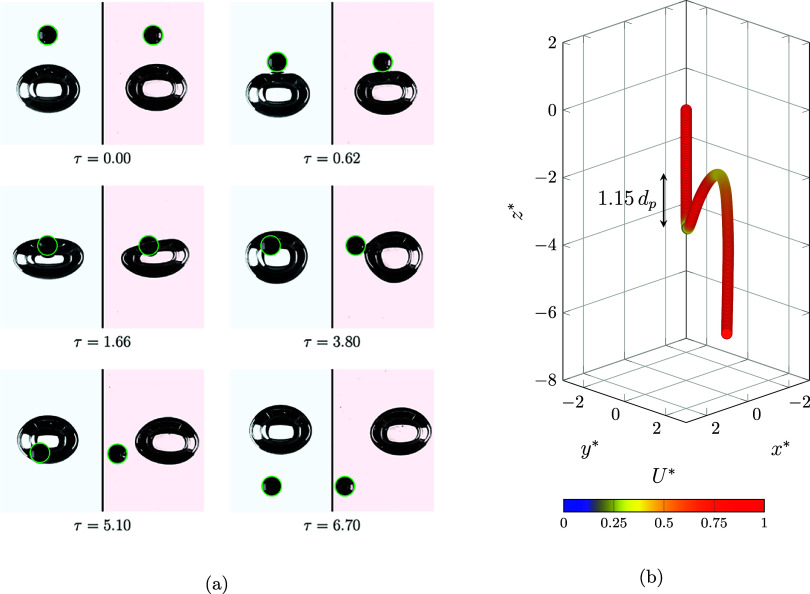
Bubble and particle interaction in the bouncing regime (*log­(Mo)* = −4.43, Γ = 6.31, λ = 0.33, and *d*_p_ = 1.5 mm). (a) Sequence image of the interaction during modified time from the front side (blue) and side view (red). (b) Extracted location of the particle center of mass during the interaction. The color bar shows the particle velocity magnitude.

The process starts, as in the previous regime, with free settling of the particle, and the velocity reaches the terminal velocity (U* = 1, Figure 7b). Then, the particle interacts with the bubble, causing significant deformation of the bubble (Figure 7a, τ = 1.66). Then, the bubble transfers the gained energy to the particle and shoots it in a side direction. After that, both the particle and bubble continued new paths after collision, until they again reached their equilibrium condition.

Variation in the particle normalized *z*-direction location and velocity magnitude is illustrated in Figure 8a. It appears that the particle continues to free-settle until τ = −1.00, while the presence of the bubble manipulates the particle motion and commences to reduce the velocity. This trend persists until τ = 1.45, after which the bubble completely stops the downward motion of the particle (U* = 0 and z* = −3.5). During 1.45 < τ < 4.45, the jumping period occurred, during which the particle is shot to one side of the bubble due to a minor initial misalignment. This shooting leads to a particle jump around 1.15 times its diameter in the opposite direction to gravity (z*) and 2.5 times in the side direction (y*) (Figure 7b). After that, the particle begins to free-settle, and its velocity approaches the terminal velocity.

**Figure 8:**
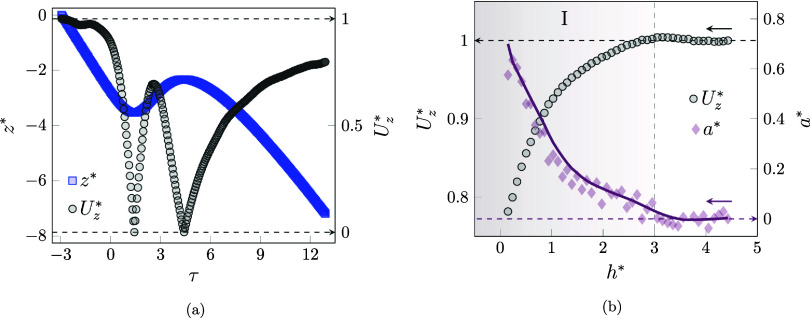
(a) Evolution of the particle normalized *z*-direction location (z*) and velocity magnitude (*U*_z_^*^) in the range of the normalized time span (τ) in the bouncing regime. (b) Variation of the particle *z*-direction velocity magnitude (*U*_z_^*^) and acceleration (a*) in the range of the minimum normalized relative distance (h*).

The behavior of the particle in the vicinity of the bubble interface before collision in the bouncing regime is demonstrated in Figure 8b. To define the variation in velocity magnitude in the *z-*direction, the normalized acceleration (a* = *a*/*g*, where *g* is gravitational acceleration) variation is included, as well. At the far end of the bubble interface, it is observed that the velocity magnitude is constant and, as a result, the acceleration is zero. When the relative distance between the bubble and particle reaches three times the particle diameter (h* = 3), the particle senses the presence of the bubble, and the velocity starts to decrease, while the acceleration increases (Stage I). Although the relative velocity between the interfaces and the particle Reynolds number are higher than in the shuttling case, the particle senses the bubble in the shorter relative distance due to the viscosity of the surrounding fluid.

Figure 9 shows the variation of the normalized hydrodynamic force over the normalized time. As the particle is in free settling and the velocity shows a narrow range, the hydrodynamic force is considered to be approximately zero, whereas at the moment of sensing the bubble, it increases. The viscous resistance of the surrounding fluid is insufficient to stop the particle completely or create a stall point before the collision. The particle collides with the interface of the bubble at τ = 0.64 and starts to semipenetrate the bubble and, as a result, deforms it.

**Figure 9:**
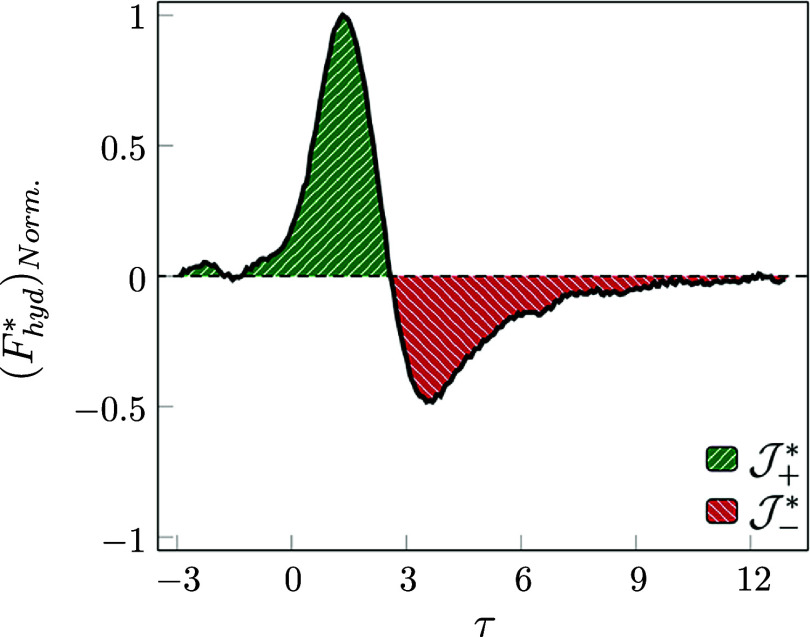
Behavior of the normalized hydrodynamic force in the bouncing regime ( $J_{+}^{*}/J_{-}^{*}=1.47$ ).

By increasing the pressure inside the bubble and the surface tension resistance relative to the kinematic energy of the particle, the particle stalls at τ = 1.45, and the increasing trend in the hydrodynamic force is halted. Then, because the bubble experienced significant deformation, the surface energy and pressure inside the bubble must be balanced; the force is then transferred back to the particle, like a spring, leading to the shooting phenomenon. In this stage, the force experienced a decreasing trend until τ = 2.67, after which the hydrodynamic force again reached the equilibrium value. After that, the particle still experienced jumping to τ = 4.45 and then again experienced free settling so that the force tends to become zero again, which is the equilibrium condition.

It is worth mentioning that by monitoring the positive and negative impulse magnitudes in this regime and comparing with the shuttling regime, the area ratio is not in the range of unity, while due to the dissipation, a portion of the energy is dissipated as viscosity, deformation, and bubble interface oscillation, and the impulse ratio is 1.47. In addition, regardless of the impulsive magnitude, the peak ratios are also different. The positive-to-negative peak ratio in the bouncing regime is around 2.29, while it is around 1.36 in the shuttling regime.

### Penetrating Regime

By maintaining the same particle relative size and density (Γ = 6.38, and λ = 0.34) and reducing the fluid viscosity (*log­(Mo)* = −5.13), the system shifted to a new regime, the penetration regime. In this regime, which is shown in Figure 10, due to the lower viscosity resistance in the fluid, the capability of the bubble to deform is greater.

**Figure 10:**
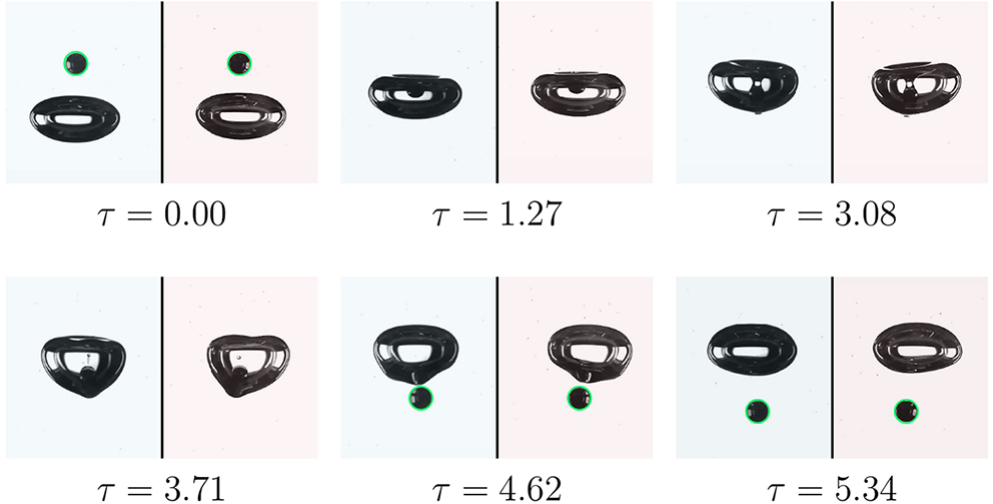
Bubble and particle interaction during the penetration regime (*log­(Mo)* = – 5.13, Γ = 6.38, λ = 0.34, and *d*_p_ = 1.5 mm).

During the collision, the lubrication force is not capable of controlling the high kinematic energy of the particle, and the surface tension force of the bubble also cannot resist the penetration of the particle. As a result, the particle invades the bubble interface, forming a transient helical throat-like tunnel (Figure 10, τ = 3.08), which shortly opens to facilitate passage and subsequently collapses once the particle exits. Unfortunately, due to the lack of optical measurements, the behavior of the particle during penetration is masked by the bubble interface.

Figure 11a illustrates the behavior of the normalized position and velocity of the particle, and Figure 11b demonstrates the variation of the hydrodynamic force during the interaction. The semistable trend of the particle velocity and steady settling, interrupted by sensing the bubble in τ = 0.8, and the velocity starts to decrease.

**Figure 11:**
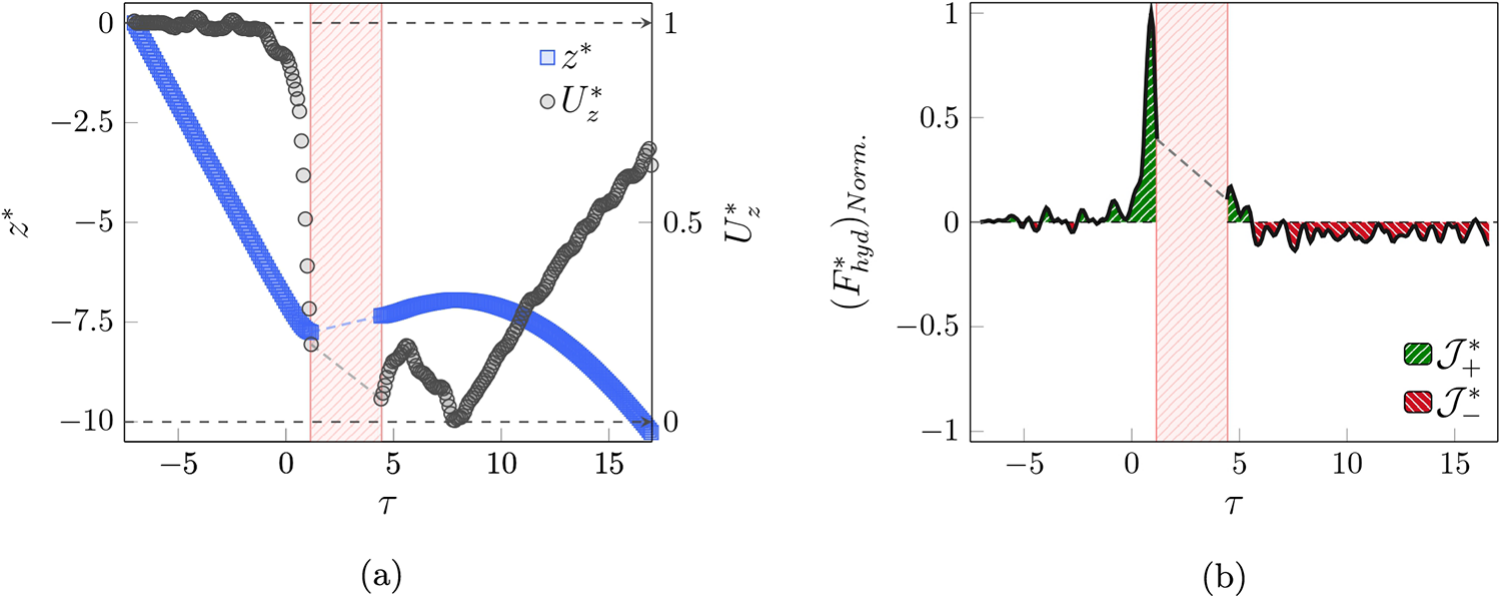
(a) Variation of the normalized location and velocity in the *z-*direction and (b) normalized hydrodynamic force over the duration of the interaction in the penetration regime.

Due to the higher inertia of the particle, the bubble interface is not able to prevent the downward motion of the particle, and as a result, the particle penetrates inside the bubble. It is worth mentioning that the velocity does not reach zero before the penetration. Additionally, the particle experiences a stall point within the bubble, and after exiting the bubble interface, it climbs for a short duration. This could be due to the forces exerted on the particle during the collision or to trapping in the vortex region below the bubble path.

The large peak before impact with the bubble interface in the force behavior is because the viscous liquid film attempts to reduce the inertia of the particle (Figure 11b). Moreover, the fluctuation after the particle exits the bubble interface is due to vortex instability beyond the bubble. Unfortunately, monitoring the behavior of the particle in this regime is not possible, and the proposed force model to describe it is incomplete due to a lack of access to the position of the particle during the interaction. In addition, the formation and behavior of the throat, as well as its stability, are other aspects of this regime that are not possible to investigate with the current method and require further study.

### Flotation Regime

The last regime is flotation, which is widely used across various industrial applications. This regime is characterized by particles attaching to the bubble interface, joining together to form a unified system, and then rising. Snapshots of the interaction and the normalized motion path are shown in Figure 12a, 12b, respectively. This regime occurs in the same fluid (*log­(Mo)* = −5.13) as the penetration regime, but with different particle properties (Γ = 5.10 and λ = 0.15). The particle settles at the bubble interface, but because of unstable attachment, it begins to slide over the bubble. However, due to the relatively small size ratio, the particle slides over one side 3.25 times its diameter. Then, by rupturing the liquid film between the bubble and particle, a three-phase contact is formed, and the particle attaches to the bubble and rises with it. Due to direct contact between the bubble and the particle, the surface wettability is another parameter that can alter the interaction; however, detailed quantification remains for future study.

**Figure 12:**
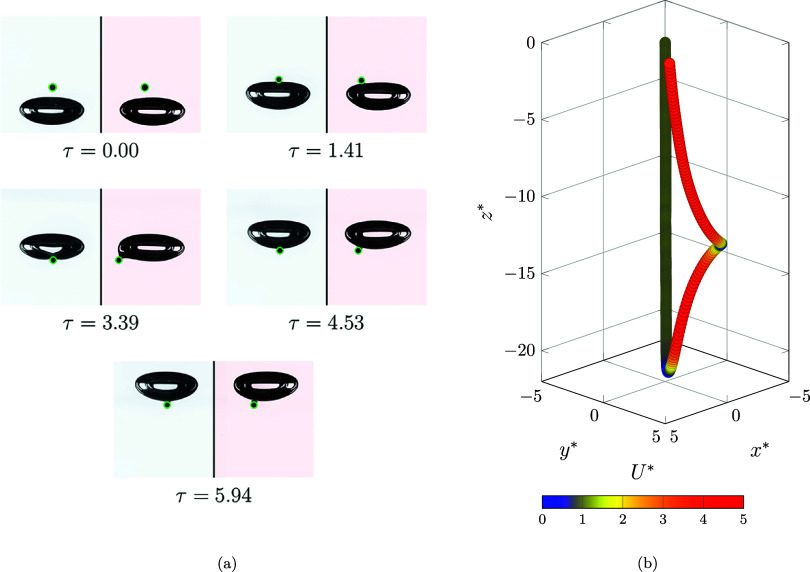
(a) Snapshots of the bubble and particle behavior in the flotation regime and (b) 3D path of the movement (*log­(Mo)* = −5.13, Γ = 5.10, λ = 0.15, and *d*_p_ = 700 μm).

The behavior of the normalized velocity components is monitored in Figure 13a. The particle settles with a constant terminal velocity, while, due to the high ratio between the bubble buoyancy and the particle weight force (|**F**_b,b_|/|**F**_g,p_| ≈ 52), the particle senses the approaching bubble in significant distance, which means the beginning of Stage I (h* = 9.42 and τ = −2.21). The reduction continues until the particle experiences a stall moment (h* = 1.57 and τ = −0.28), after which the particle starts to climb and attempts to match the bubble velocity (Stage II). The particle then settles on the bubble at τ = 0.45, end of the second stage, and the velocity climbs rapidly; at τ = 1.08, it reaches the bubble velocity (*U*_z_^*^ = 4.13).

**Figure 13:**
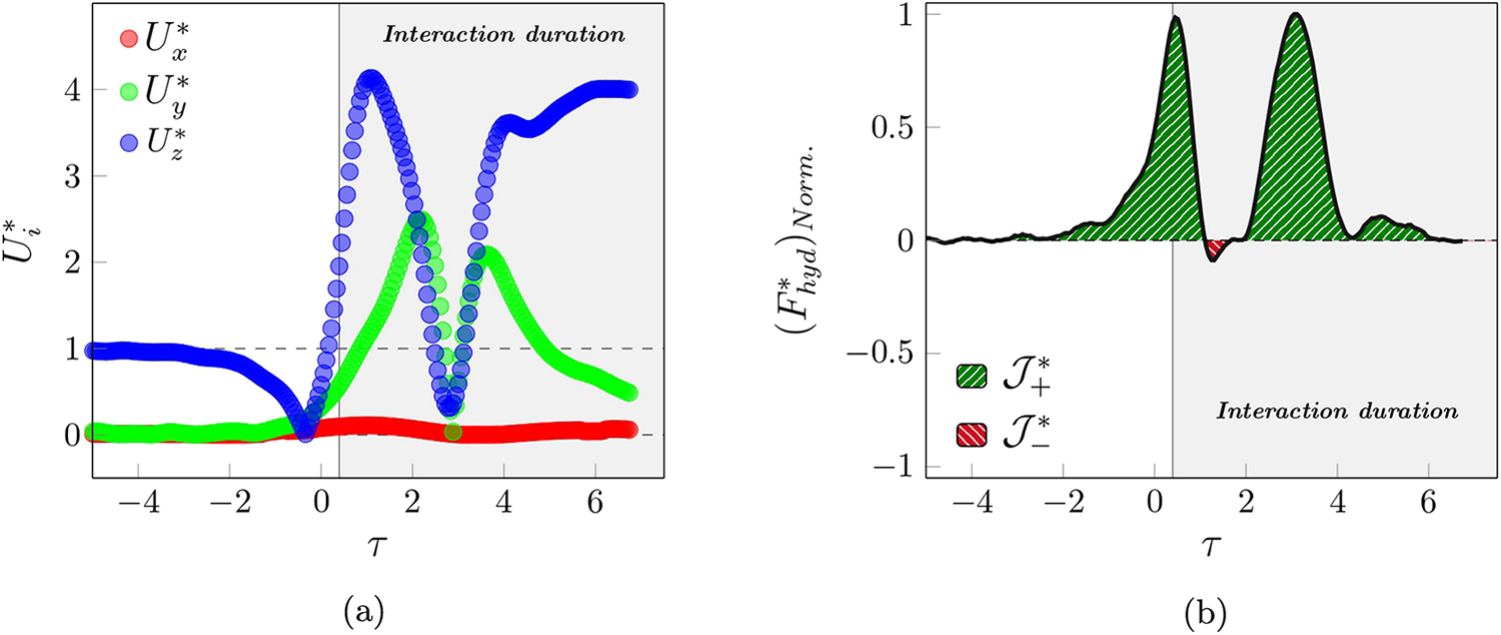
(a) Variation of the normalized particle velocity component and (b) behavior of the normalized hydrodynamic force over the flotation regime.

After this time, the particle starts to slide on one side of the bubble, and the *U*_y_^*^ component begins to increase, while the *U*_z_^*^ component experiences a reduction. Thereafter, instead of sliding over the bubble and moving farther from the bubble interface, the particle attached to it again reached a stall point (τ = 2.89). Then, *U*_y_^*^ decreases to again become zero, while *U*_z_^*^ starts to increase to reach the new transfer velocity of the bubble and attached particle (*U*_z_^*^ = 4.01).

The variation of the hydrodynamic force is demonstrated in Figure 13b. The behavior of the force, as in other regimes, starts with a positive impulse. As the bubble begins to impede the downward motion of the particle, the force increases to τ = −2.21. The force peaks at τ = 0.45 and then decreases as it approaches the bubble velocity, until τ = 1.08, which reaches zero. As the particle-to-bubble size ratio is too small, the negative impulse is not observed because the sliding phenomenon is not the same as in previous regimes. In addition, by starting the rotation over the bubble, a second positive impulse occurred, and after that, as the particle matched the velocity of the bubble, the hydrodynamic force tended to reach a stable value. It is worth mentioning that, due to the nature of the flotation process, the impulse ratio is significantly greater than 1, i.e., $J_{+}^{*}/J_{-}^{*}≃O$ ­(10).

## Conclusions

The behavior of a freely settling particle and a rising bubble in a range of fluids is investigated experimentally. A small-scale setup is developed to identify and characterize the complex nature of this interaction. The 3D path of the particle is reconstructed from high-speed photography. To overcome the limitations of conventional image processing methods for identifying the particle location, a new multioperational approach is developed to prevent errors, especially when the bubble and particle mask each other during interaction.

Based on the time-dependent extracted particle location, a hydrodynamic force and impulse investigation is conducted. Four different interaction outcomes are specified based on the behavior of the particle. At a higher Morton number (*log­(Mo)* = −2.71), the shuttling regime occurs, as the viscosity of the surrounding fluid and the approaching bubble can stop the downward motion of the particle and shuttle it against gravity. At a moderate Morton number and high density ratio (*log­(Mo)* = −4.43, Γ = 6.31), the inertia force of the particle dominates the fluid resistance. At the same time, the surface tension force remains an important parameter controlling the settling of the particle and, instead, leading to the named bouncing regime. However, at a lower Morton number (*log­(Mo)* = −5.13), the behavior of the interaction is more related to the density ratio and size ratio. At a higher density ratio (Γ = 6.38), the surface tension is unable to overcome the inertia of the particle, and the particle penetrates the bubble interface by forming a transient helical, throat-like tunnel. This regime is defined as a penetration regime. If the particle size ratio is too small (λ = 0.15) in comparison with the bubble size, then, the flotation regime emerges, during which the particle is attached to the bubble, and a single rising system occurs. This demonstrated that the Morton number is not the only parameter to characterize the mentioned regime, and future investigation is required. The proposed approach has the potential to identify the nature of the applied force in the interaction. It should be noted that the four regimes identified in this study serve as a general classification framework within which additional subregimes or transitional behaviors can potentially be defined based on variations in system parameters, including particle–bubble alignment and fluid viscosity range.

This small-scale study addresses the characteristics of bubble–particle interactions. It offers insights that can provide an initial understanding of physics-based upscaling methods for simulating large-scale scenarios. Future work should also extend this study to identify the transition between bubble–particle interaction regimes across a range of physical parameters relevant to industrial-scale slurry bubble columns and to construct a corresponding regime map.
